# Magnetofection and isolation of DNA using polyethyleneimine functionalized magnetic iron oxide nanoparticles

**DOI:** 10.1098/rsos.181369

**Published:** 2018-12-12

**Authors:** Adheesha N. Danthanarayana, Danushika C. Manatunga, Rohini M. De Silva, N. Vishvanath Chandrasekharan, K. M. Nalin De Silva

**Affiliations:** 1 Department of Chemistry, University of Colombo, Colombo 00300, Sri Lanka; 2 Sri Lanka Institute of Nanotechnology (SLINTEC), Mahenwatta, Pitipana, Homagama 10200, Sri Lanka

**Keywords:** iron oxide nanoparticles, polyethyleneimine, DNA isolation, magnetofection

## Abstract

This study was carried out to develop a simple and efficient method to isolate DNA directly from biological samples using iron oxide nanoparticles (IONPs) functionalized with polyethyleneimine (PEI). IONPs were synthesized via co-precipitation method followed with direct attachment of branched PEI. Nanoparticles were characterized using STEM, FT-IR spectroscopy and XRD analysis. The binding capacity of synthesized PEI-IONPs for plasmid and genomic DNA was assessed using purified DNA samples. In order to elute bound DNA, elution conditions were optimized, changing pH, salt concentration and temperature. Synthesized PEI-IONPs were subjected to isolation of DNA from bacterial cell culture and from human blood. PCR and magnetofection of the enhanced green fluorescence protein (EGFP) were carried out to verify the downstream applications of isolated DNA. The results indicated that the synthesized nanoparticles were of 5–10 nm. The binding capacity of PEI-IONPs for plasmid DNA and genomic DNA were 5.4 and 8.4 µg mg^−1^, respectively, which were even higher than the commercially available kits such as Mag-bind, MagJET and Magmax. The optimized condition for plasmid DNA elution was 0.1 M Tris HCl (pH 10.0), 1.5 M NaCl and 5% formamide, maintained at the temperature of 60°C. The optimized condition for genomic DNA elution was 0.1 M Tris HCl (pH 10.0), 1.5 M NaCl and 10% formamide, maintained at 60°C. PCR and magnetofection processes were successful. This study revealed that the magnetic separation of DNA using PEI-IONPs is a simple and efficient method for direct isolation of DNA from biological samples which can be then used in various downstream applications.

## 1. Introduction

Efficient methods of DNA isolation is an ever growing subject in much research, as purified DNA is extremely important in medical and biotechnological fields. As far as existing methods are concerned, chromatographic methods [1], organic extraction methods [2], anion exchange methods [2] and salting-out methods [2] are most commonly used. Nevertheless, there are so many drawbacks such as time consumption, use of toxic chemicals and the need of specialized equipment [3]. In the recent past, the development of magnetic purification methods has attracted much attention, and in this context iron oxide nanoparticles are the most extensively studied due to their unique properties such as lower toxicity, biocompatibility and easy degradability. Therefore, iron oxide nanoparticles have permitted most suitable and safest nanomaterial for many *in vivo* applications including magnetic resonance imaging (MRI) [4–6], drug and gene delivery [5,7–9], bioseparation [10] and hyperthermia [5,11].

Iron oxide nanoparticles are composed of nanocrystallites of magnetite (Fe_3_O_4_) or maghemite (γ-Fe_2_O_3_) [12]. Even though magnetite possesses ferromagnetic properties, if the size of the nanoparticles is very small (10–20 nm), they exhibit superparamagnetic properties [12]. As a result, the bio-distribution of nanoparticles can be altered by applying an external magnetic field, and therefore they are widely used for biomedical applications specially due to their dispersity without forming magnetic aggregates.

In addition, the functionalization of iron oxide nanoparticles has been well studied with surfactants, polymers, biomolecules and also with inorganic layers such as silica or metal [6,13]. Additionally, mono-coated nanoparticles can be further coated to improve their targeted task. Cetyltrimethyl ammonium bromide (CTAB) [14,15], one of the most common surfactants used in synthesis of iron oxide nanoparticles, can be strongly adsorbed onto the surface of iron oxide nanoparticles through its head group and thereby it forms micelles in aqueous solutions making the nanoparticles water soluble and monodispersed [13]. Polyethyleneimine (PEI) is a cationic polymer which can form strong ionic interactions with negatively charged phosphate backbone of DNA [3,16] and some of the proteins [17]. Therefore, it has been paid great attention to use PEI functionalized magnetic iron oxide nanoparticles for isolation of DNA as there are many advantages, for instance, the technique is simple and rapid compared with the conventional DNA isolation methods [18]. Additionally, it eliminates the need for costly equipment as well as the use of toxic chemicals [19]. Furthermore, as the quantity is small and reusable it has allowed the large-scale purification [3]. It is also revealed that the DNA isolated using PEI functionalized iron oxide nanoparticles has been used directly in downstream applications. According to the work done by Chiang *et al*., plasmid DNA isolated using PEI functionalized iron oxide nanoparticles has been used directly for restriction of enzyme digestion, bacterial cell transformation and animal cell transfection [3]. According to the published data, it is worth noticing that PEI functionalized iron oxide nanoparticles have not been used to isolate genomic DNA directly from biological samples [20–22]. However, PEI functionalized nanoparticles have been successfully used as agents for DNA transfection via magnetofection technique in previous studies [23–25]. Therefore, in this study, PEI functionalized iron oxide nanoparticles were subjected to isolate genomic DNA from biological samples, which can be directly used in several downstream applications such as polymerase chain reaction. In addition to DNA isolation, it was also attempted to increase the gene transfection efficiency of the isolated plasmid DNA mainly by reducing the incubation time.

## 2. Material and methods

### 2.1. Chemical reagents

Ammonium iron(III) sulphate dodecahydrate (NH_4_Fe(SO_4_)_2_.12H_2_O), branched polyethyleneimine (Avg. M.W. ∼ 25 000), cetyltrimethyl ammonium bromide (CTAB), formamide and agarose were purchased from Sigma-Aldrich. Ammonium iron(II) sulphate hexahydrate ((NH_4_)_2_Fe(SO_4_)_2_.6H_2_O), aqueous NH_3_ solution (purity = 30% w/w) and ethanol were purchased from MERCK. Phosphate saline buffer (PBS, pH 7.5), Tris EDTA (TE buffer, pH 7.5) which was used as the binding buffer and washing buffer, 0.5x Tris borate EDTA (TBE), gel loading buffer, diluted ethidium bromide solution, ET buffer, lysis buffer (10 N NaOH, 20% SDS, autoclaved water), KAc solution, RBC lysis buffer, LB 1 buffer, LB 2 buffer, DB buffer and pcDNA 3.1 (5.4 kb) having the EGFP gene (Invitrogen) which was cloned in XL1-Blue *E. coli* were supplied by the Molecular Biology Laboratory in University of Colombo. Elution buffers (which contained Tris HCl, NaCl and autoclaved water) were prepared using molecular biology grade chemicals. Molecular biology grade LB agar was purchased from Lennox. MEM (Eagle's minimum essential medium—M0769), 10% FBS (fetal bovine serum—F2442), NaHCO_3_ (S5761), penicillin-streptomycin solution (P4333), L-glutamine (49419), trypsin (T4799) and EDTA (ethylene diamine tetra acetate—E6758) were purchased from Sigma-Aldrich. Preserved HEp-2 cell vial was provided by the Biochemistry Department of Faculty of Medicine, University of Colombo. Distilled autoclaved water was used in the media preparation. Double distilled, degassed water was used for all the experiments.

### 2.2. Preparation of PEI functionalized iron oxide nanoparticles

#### 2.2.1. Synthesis of iron oxide (Fe_3_O_4_) nanoparticles (IONPs)

The co-precipitation method was used for the synthesis of (Fe_3_O_4_) nanoparticles. First, a solution of 0.1 M Fe(III) (100.00 ml) and a solution of 0.1 M Fe(II) (50.00 ml) were degassed in an inert environment for about 30 min. A solution of 5.0 M NH_4_OH (20.0 ml) was added drop-wise into this mixture containing 0.1 M CTAB (25.0 ml) over a period of about 30 min under N_2_ until the pH of the medium approached 12. The resulting black slurry was separated, washed and dried in a vacuum desiccator. The product was characterized using FT-IR analysis.

#### 2.2.2. Functionalization of iron oxide nanoparticles with polyethyleneimine (PEI-IONPs)

A 20% (w/w) PEI solution (8.5 ml) was prepared using distilled ethanol and it was added to a solution of 5 M NH_4_OH (20.0 ml). Then the mixture was stirred at 50**°**C using a magnetic stirrer. After about 5 min, 0.80 g of pre-synthesized iron oxide nanoparticles was added and stirring was continued for another 30 min. The resulting slurry was separated using a magnet and the pellet was washed with double distilled water several times. A portion of the pellet was dried and was characterized using FT-IR spectroscopy (Bruker Vertex 80), X-ray diffraction (XRD; CuKα = 1.5418 Å, Bruker D8 Focus X-Ray diffractometer) and scanning transmission electron microscopy (STEM—Technai G2 F20, FEI Company).

### 2.3. Application of PEI-IONPs for DNA isolation

#### 2.3.1. Determination of plasmid DNA and genomic DNA binding capacity of PEI-IONPs

PEI coated iron oxide nanoparticles (5 mg) were suspended in 100 µl of TE buffer for 2 min and the supernatant was removed by magnetic separation. This washing step was repeated once. Then again 100 µl of TE buffer was added to these nanoparticles which were then incubated with 5.0 µl of plasmid DNA (1 µg µl^−1^). It was shaken for 15 min at room temperature and then an aliquot of 10.0 µl of the supernatant was collected by magnetic separation. To the same initial sample, 5.0 µl of plasmid DNA and 5.0 µl of TE buffer were added and it was mixed for 15 min. After applying the magnetic stand, an aliquot of 10.0 µl of the supernatant was collected. Likewise, the procedure was repeated several times to collect the supernatants separately after adding plasmid DNA and TE buffer each time.

A volume of 5.0 µl of each separated supernatant was mixed with 2 µl of gel loading buffer and loaded onto a 0.8% agarose gel. Gel electrophoresis was carried out for about 30 min at 80 V and checked for a DNA band. The supernatants were collected until a DNA band was seen on the gel and the total amount of DNA added was noted down. This procedure was carried out to get an idea about the approximate range of binding capacity. In order to determine the exact value of binding capacity, the obtained range was expanded by collecting supernatants separately after adding 1.0 µl of plasmid DNA and 9.0 µl of TE buffer each time. In addition to determine the genomic DNA binding capacity, the same procedure was carried out using genomic DNA instead of plasmid DNA.

#### 2.3.2. Elution of purified plasmid DNA and genomic DNA from PEI-IONPs

A volume of 100 µl of TE was added to 5 mg of nanoparticles which was then incubated with 15 µl of plasmid DNA (1 µg µl^−1^). It was shaken for 15 min and then the supernatant was collected. A volume of 100 µl of TE was added to the plasmid DNA bound nanoparticles and it was mixed for 5 min and the supernatant was collected. This washing step was repeated once and the supernatants were collected to ensure that the bound plasmid DNA was not eluted during the washings. In order to elute the DNA from the nanoparticles, 100 µl of elution buffer was added to the nanoparticles bound with DNA and incubated for 15 min and then the supernatant with eluted DNA was collected. The same step was repeated to remove the entire DNA that was bound to nanoparticles. All the supernatants during washing and elution steps were collected separately and gel electrophoresis was carried out at 80 V to check for the DNA bands.

For the elution of genomic DNA, the same procedure was carried out using 6.0 µl of genomic DNA (2.5 µg µl^−1^) instead of plasmid DNA.

#### 2.3.3. Optimization of elution conditions

The above procedure was repeated using different elution buffers having different salt concentrations and pH values and also at different elution temperatures (from 30°C to 70°C) to find the optimum conditions for elution of plasmid DNA and genomic DNA.

#### 2.3.4. Isolation of DNA from biological samples

##### 2.3.4.1. Preparation of bacterial cell lysate and isolation of plasmid DNA using PEI-IONPs

A volume of 1.0 ml of the bacterial cell culture (XL1-Blue *E. coli*) prepared using LB agar medium was poured into an Eppendorf tube and it was centrifuged at 12 000 r.p.m. for 12 min to obtain the bacterial pellet. The resulting pellet was re-suspended in 150 µl of ET buffer (which mainly contains EDTA), and treated with RNase (20 mg ml^−1^) followed with the lysis buffer (10 N NaOH, 20% SDS, autoclaved water) and potassium acetate. After keeping it in ice for 15 min the bacterial cell lysate was centrifuged at 14 000 r.p.m. for 5 min. The resulting supernatant was carefully transferred to a new tube. Half of the collected supernatant was separated to use as the control. The other half was used to bind with nanoparticles.

To 5.0 mg of nanoparticles, 40.0 µl of the supernatant with 100 µl of TE buffer was added. It was mixed well for about 15 min and the supernatant was collected using the magnetic stand. A volume of 100 µl of the elution buffer (optimized buffer) was added to DNA bound nanoparticles. It was mixed for 15 min and kept in a 60°C water bath (optimized temperature) for about 15 min. The supernatant with eluted DNA was collected. From each of the collected supernatant, a volume of 5 µl was run on a 0.8% agarose gel. Gel electrophoresis was carried out for about 30 min at 80 V to check for the DNA bands.

##### 2.3.4.2. Preparation of a proteinase K treated human blood sample and isolation of genomic DNA using PEI-IONPs

To a volume of 100 µl of fresh human blood sample, 600 µl of RBC lysis buffer was added and mixed well. It was centrifuged and to the pellet a volume of 250 µl of RBC lysis buffer was added again and mixed well. It was centrifuged and then a volume of 25 µl of LB 1 buffer was added and it was incubated for 15 min at 55°C. After that, a volume of 75 µl of LB 2 buffer and 5 µl of DB buffer were added and mixed well. It was incubated for two hours at 55°C. The supernatant was collected. Half of the collected supernatant was separated to use as the control. The other half was used to bind with nanoparticles.

To a volume of 50 µl of the supernatant of the proteinase K treated human blood sample, 5 mg of nanoparticles and 100 µl of TE buffer were added. It was mixed for 15 min and the supernatant was collected using a magnetic stand. Finally, 100 µl of elution buffer (optimized buffer) was added. It was mixed for 15 min and heated up to 60°C (optimized temperature) by keeping in a water bath for about 15 min. The supernatant with eluted DNA was collected. Each collected supernatant was run on a 0.8% agarose gel. Gel electrophoresis was carried out for about 30 min at 80 V to check for the DNA bands.

### 2.4. Downstream applications of DNA isolated from biological samples

#### 2.4.1. Polymerase chain reaction (PCR) for isolated genomic DNA

The region of exon 3 (560 bp) in Interferon regulatory factor 6 (IRF 6) gene was amplified using the polymerase chain reaction. The test samples and the negative control were prepared according to table 1. Reagents were mixed as in the order given. Two test samples were prepared using the supernatant of the proteinase K treated blood sample. The supernatant before binding was added to the first test sample and the supernatant after elution was added to the second test sample as the templates. PCR was carried out for 35 cycles under the optimized conditions (for a template of 12.5 ng µl^−1^) given in electronic supplementary material, table S1. After the completion of PCR, a volume of 5.0 µl of each test sample, negative control and the marker (500 bp) were loaded onto a 0.8% agarose gel and gel electrophoresis was carried out at 80 V for about 30 min.

**Table 1.** Preparation of reaction mixtures for PCR.

reagents	test sample volumes (μl)	negative control volumes (μl)
(1) PCR water	13.2	14.5
(2) Master mix	7.5	7.5
(3) forward primer (VE3F)	1.0	1.0
(4) reverse primer (VE3R)	1.0	1.0
(5) genomic DNA	1.3	—
(6) Taq polymerase	1.0	1.0
total volume	25.0	25.0

#### 2.4.2. Magnetofection of enhanced green fluorescence protein (EGFP) into HEp-2 cells

##### 2.4.2.1. Isolation of plasmid DNA using PEI-IONPs

In the provided sample, pcDNA 3.1 (5.4 kb) having the EGFP gene was cloned in XL1-Blue *E. coli*. Plasmid DNA in that given XL1-Blue *E. coli* culture was isolated using PEI coated iron oxide nanoparticles using the same procedure explained in §2.3.4.1.

##### 2.4.2.2. Culturing of HEp-2 cells

Cells were cultured in a MEM medium with 10% FBS, 3% glutamine, penicillin-streptomycin and 7.7% NaHCO_3_ at 37°C for 2–3 days until a monolayer was formed (85%–90% confluency). Daily observations were taken using the inverted fluorescence microscope with 2 MP digital camera (1×70-S1F2, Olympus, MDC 200 microscope digital camera). Cells were split using trypsin EDTA and seeded at 1.25 × 10^5^ cells per well in a 24 cell well plate (142475, Nunc Multidishes, NunclonTM, Thermo Scientific) for 24 h prior to the transfection process.

##### 2.4.2.3. Use of PEI-IONPs for gene transfection

A 1 µg µl^−1^ suspension of iron oxide nanoparticles was prepared using phosphate saline buffer (pH 7.5). Then magnetofection was carried out after mixing the reagents as given in table 2.

**Table 2.** Amount of reagents mixed for each system for transfection of HEp-2 cells.

reagents	test sample (µl)	control 1 (µl)	control 2 (µl)
amount of pcDNA (550 ng µl^−1^)	3.7	3.7	0.0
amount of PEI-IONP (1 µg µl^−1^)	10.0	—	—
amount of PEI (1000×) 20%	—	25.0	—
amount of MEM	186.3	171.3	200
total amount (µl)	200	200	200

DNA and other reagents were mixed to form the test sample and controls and incubated for 20 min at room temperature. Prior to the transfection, the medium in the wells was removed keeping the cells in the wells. Then these cells were washed with PBS for twice and each well was replaced with 200 µl of incomplete medium (medium lacking 10% FBS) and was allowed to stand for 15 min at 37°C. After 20 min of incubation of the prepared nanoparticle systems, 200 µl from each system was added to the cell wells. This was triplicated. After that the cell well plate was placed on a magnetic box containing permanent magnets (NdFeB) having field strength of 150 mT. Magnetic field was supplied over a period of 15–20 min and later medium in each well was removed and was replaced with 200 µl of complete medium containing 10% FBS. Then the cells were incubated at 37°C for 24 h. After 24 h of incubation, the medium in the wells was removed and PBS was added to each well. It was examined under the fluorescence microscope to check for the expression of green fluorescence protein.

## 3. Results

### 3.1. Characterization of iron oxide nanoparticles

FT-IR spectroscopy was carried out to confirm the formation of CTAB coated iron oxide nanoparticles and the spectrum is given in electronic supplementary material, figure S1. The peak at 582 cm^−1^ represents the Fe–O band vibration of iron oxide nanoparticles [14]. The peaks at 3368 cm^−1^ and 1626 cm^−1^ correspond to O–H stretching and bending bands of iron oxide nanoparticles [26]. The peak at 1382 cm^−1^ is due to the C–N bond of CTAB. The peak around 1450 cm^−1^ is related to CH_2_ scissoring vibrations and the peaks in the range of 2800–3000 cm^−1^ are due to CH_2_ stretching vibrations of CTAB and these peaks are in accordance with the previous studies [14,27].

**Figure 1.**
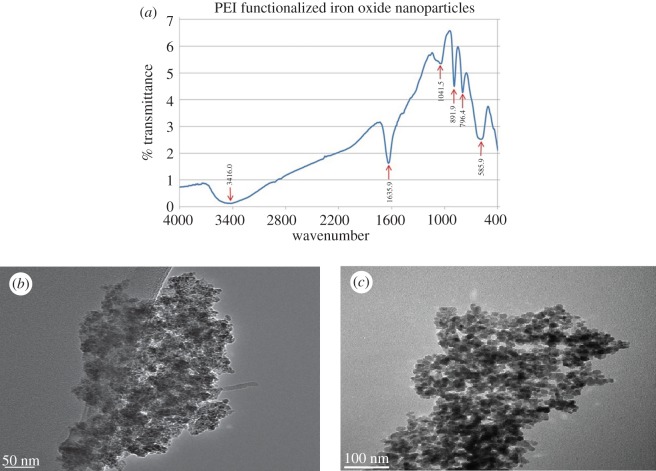
(*a*) FT-IR spectrum of polyethyleneimine functionalized iron oxide nanoparticles, (*b*) STEM image of PEI coated iron oxide nanoparticles and (*c*) TEM image of neat IONPs.

CTAB coated iron oxide nanoparticles were then subjected to functionalization with PEI. The FT-IR spectrum obtained for PEI functionalized iron oxide nanoparticles is given in figure 1*a*. The peak at 3416 cm^−1^ is due to the -NH stretching vibrations of PEI. The characteristic, distinct peak of -NH scissoring vibrations of PEI can be seen at 1635 cm^−1^ and the peak around 1041 cm^−1^ corresponds to the weak CN stretching vibrations of PEI. These peaks are in good agreement with the previously reported results [28]. In addition, it was clear that the ligand CTAB has been replaced by PEI.

Size and the morphology of the PEI coated iron oxide nanoparticles were studied using scanning transmission electron microscope (STEM) and the STEM image obtained is given in figure 1*b*. When compared with the image of the neat IONPs which is given in figure 1*c*, it is clearly understood that the size of the particles has increased with the polymer coating. The exact size was hard to determine as the particles were embedded in a mesh of the polymer PEI and they seem to have a diameter of 20 nm. Also, these particles were trapped in the polymer net of PEI where each of the iron oxide nanoparticles was bound with more than one strand of PEI, as PEI is known to be a branched polymer. This bridging aggregation is in accordance with the results reported in the work done by Petri-Fink *et al.* [29].

**Figure 2.**
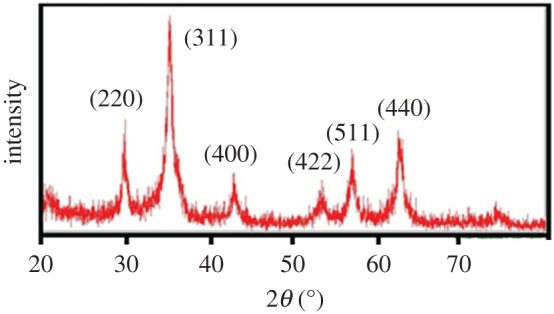
XRD pattern of PEI functionalized iron oxide nanoparticles.

X-ray diffraction (XRD) was performed to obtain the crystalline structure of the synthesized nanoparticles. The XRD pattern for PEI functionalized iron oxide nanoparticles is given in figure 2. The six characteristic peaks for Fe_3_O_4_ at 2*θ* = 30.1**°**, 35.5**°**, 43.1**°**, 53.4**°**, 57.0**°** and 62.6**°** indicated that the synthesized particles are Fe_3_O_4_ with a cubic inverse spinel structure [3].

### 3.2. Determination of plasmid DNA and genomic DNA binding capacity of PEI functionalized iron oxide nanoparticles

PEI coated iron oxide nanoparticles were then used to assess the DNA binding capacity of both plasmid and genomic DNA. Purified DNA samples were used for this experiment.

#### 3.2.1. Plasmid DNA binding capacity

Synthesized PEI coated iron oxide nanoparticles were mixed with plasmid DNA as given in the §2.3.1 and the binding capacity was assessed using the gel electrophoresis as given in electronic supplementary material, figure S2.

**Figure 3.**
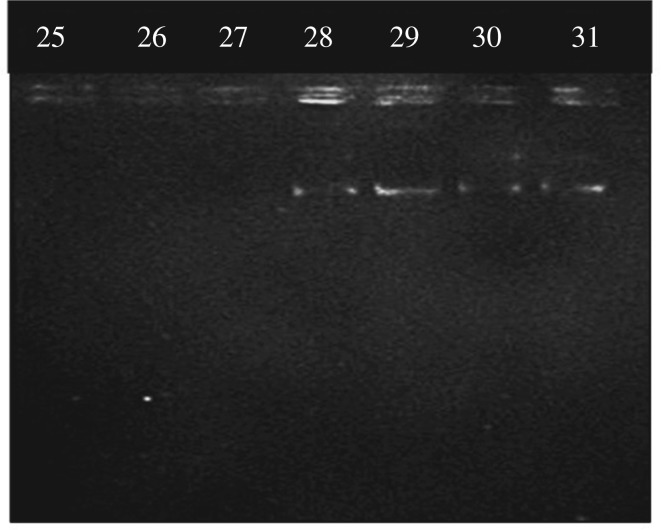
Gel picture of plasmid DNA remaining in the supernatant when the added DNA content is in the range of 25–31 µg.

In the gel picture given in electronic supplementary material, figure S2, the first well contained the control and the other six wells contained the supernatants after adding DNA. Until up to the amount of 25 µg of plasmid DNA, no DNA was found in supernatant. The presence of DNA in the supernatant was found only after the addition of 30 µg of plasmid DNA. In order to find the exact plasmid DNA binding capacity, the DNA content was ranged between 25 and 31 µg. The gel picture obtained for this experiment is shown in figure 3.

From this gel picture, the presence of DNA band can be seen at the amount of 28 µg and therefore it was clear that the maximum amount of plasmid DNA that can be bound by 5 mg of nanoparticles was 27 µg. Therefore, the plasmid DNA binding capacity of PEI functionalized iron oxide nanoparticles was 5.4 µg mg^−1^ of nanoparticles.

#### 3.2.2. Genomic DNA binding capacity

An amount of 5 mg of synthesized PEI coated iron oxide nanoparticles was initially mixed with 15 µl of genomic DNA (1 µg µl^−1^) and the experiment was carried out the same as for plasmid DNA. The added DNA content was varied from 15 to 50 µg. The gel picture obtained is given in electronic supplementary material, figure S3.

**Figure 4.**
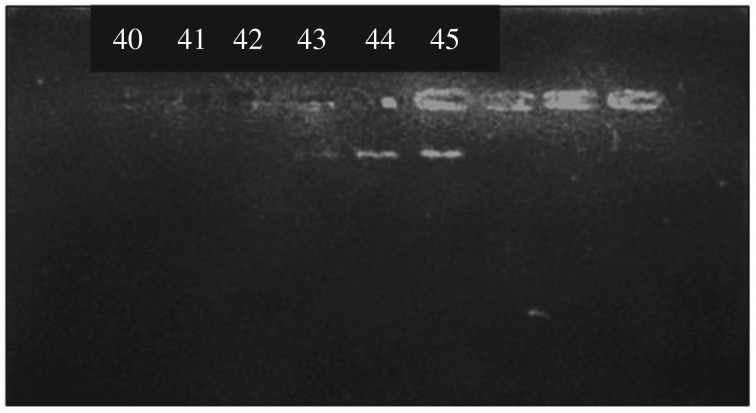
Gel picture of the remaining genomic DNA in the supernatant under optimized conditions.

As in electronic supplementary material, figure S3, the presence of DNA bands was seen after the addition of 45 µg of genomic DNA. To find the exact genomic DNA binding capacity the experiment was again carried out, in the range of 40–45 µg and the resulted gel picture is given figure 4.

According to the gel picture, it is clear that there is a faint band of DNA at 43 µg. Therefore, the genomic DNA binding capacity was found to be 8.4 µg mg^−1^ of nanoparticles.

### 3.3. Elution of purified plasmid DNA and genomic DNA and optimization of elution conditions

#### 3.3.1. Elution of purified plasmid DNA

In order to find the optimum elution conditions, the elution was carried out using different elution buffers having different salt concentrations and pH values and also at different temperatures, as given in §§2.3.2 and 2.3.3 in detail.

First, the elution buffer system was optimized. However, the first buffer system (elution buffer 1 containing 0.1 M Tris HCl (pH 9.0), 1.25 M NaCl) to be tested was already reported [3]. But, elution was not successful, as shown in gel picture in electronic supplementary material, figure S4.

According to this gel picture, a DNA band was seen only for the control. No DNA band for the supernatant implies that all the DNA has been bound with PEI-IONPs. The absence of DNA bands in washings implies that no DNA eluted during the washings which confirms the strong binding. The elution buffer was added to the DNA bound PEI-IONPs to see if DNA can be eluted. However, electronic supplementary material, figure S4 shows there are no DNA bands corresponding to the wells 5, 6 and 7. This implies that DNA was bound well with nanoparticles and they were not eluted properly when elution buffer 1 was used. Therefore, the elution was not successful with the reported elution buffer at room temperature.

By weakening the electrostatic interactions between DNA and PEI, it was expected that elution of DNA can be performed. In order to do that, the temperature of the system was raised to 60**°**C with the same buffer system. The gel picture obtained for this experiment is given in electronic supplementary material, figure S5.

According to the gel picture, again no DNA bands eluted under this condition. Therefore, the new elution buffer system (elution buffer 2 containing 0.1 M Tris HCl (pH 10.0) and 1.5 M NaCl) was prepared by increasing the pH value and the salt concentration in order to destabilize DNA–PEI complexes [17]. The elution temperature was maintained at 60**°**C as same as the previous experiment. The gel picture obtained is given in electronic supplementary material, figure S6.

**Figure 5.**
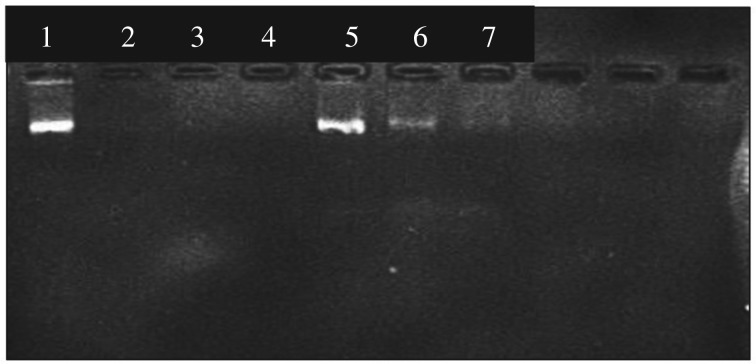
Gel picture of plasmid DNA eluted with elution buffer 3 provided with heat at 60**°**C (1) control, (2) supernatant after binding DNA, (3) washing 1, (4) washing 2, (5) elution 1, (6) elution 2, (7) elution 3.

According to electronic supplementary material, figure S6, there was a clear DNA band for the elution 1 and a very faint band was seen for the elution 2. However, when comparing the intensities of the bands of the control and elutions, the recovery was not very successful. Therefore, aiming to increase the recovery, another buffer system (elution buffer 3) was prepared by adding 5% formamide while keeping the other conditions the same as the previous experiment. The resulted gel picture for this buffer system is given in figure 5.

According to this gel picture, there is a strong intense DNA band at the fifth well corresponding to elution 1. Therefore, it can be evidenced that most of the bound DNA has been eluted in elution 1, suggesting that elution was successful with elution buffer 3.

The same buffer system with formamide was then used to obtain the optimum temperature, as given in §2.3.3. There, the temperature was varied in the range of 30°C–70°C and the obtained gel picture is given in electronic supplementary material, figure S7.

As shown in electronic supplementary material, figure S7, the highest intensity DNA band was obtained for the elution at 60**°**C. Therefore, 60**°**C was selected as the optimum temperature for the elution. Considering the above results, an elution buffer with 0.1 M Tris HCl, 1.5 M NaCl and 5% formamide, which has a pH of 10.0 and an elution temperature of 60**°**C was identified as the optimum conditions for the plasmid DNA elution.

#### 3.3.2. Elution of purified genomic DNA

Genomic DNA bound PEI-IONPs were subjected to the elution using elution buffer 3 at 60°C (optimized conditions for plasmid DNA elution) as described in §2.3.2. The resulting gel picture is given in electronic supplementary material, figure S8.

**Figure 6.**
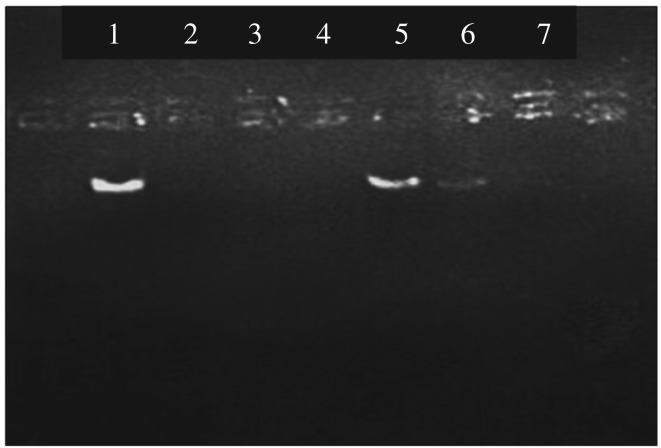
Gel picture of genomic DNA elution with elution buffer 4 heated up to 60**°**C (1) control, (2) supernatant after binding DNA, (3) washing 1, (4) washing 2, (5) elution 1, (6) elution 2, (7) elution 3.

By looking at the gel picture, it is clear that there is only a very faint DNA band corresponding to elution 1. The reason for this can be attributed to the stronger interaction between PEI and genomic DNA than PEI and plasmid DNA. In order to elute genomic DNA, a new buffer, system 4 (which contained 0.1 M Tris HCl (pH 10.0), 1.5 M NaCl, 10% formamide) was prepared by increasing formamide concentration up to 10%. Elution was carried out at 60**°**C and the gel picture obtained is given in figure 6.

In this gel picture, the intense DNA band corresponding to elution 1 shows that most of the bound DNA has eluted with elution buffer 4 indicating that DNA elution was successful with the elution buffer 4. According to the above results, an elution buffer with 0.1 M Tris HCl, 1.5 M NaCl and 10% formamide, which has a pH of 10.0 and an elution temperature of 60**°**C was identified as the optimum conditions for genomic DNA elution.

### 3.4. Isolation of DNA from biological samples

#### 3.4.1. Isolation of plasmid DNA from a bacterial cell culture

**Figure 7.**
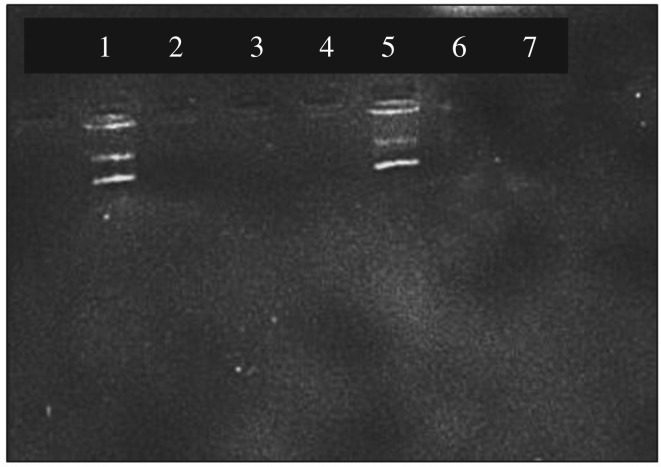
Gel picture of plasmid DNA eluted from PEI-IONPs (1) control, (2) supernatant after binding DNA, (3) washing 1, (4) washing 2, (5) elution 1, (6) elution 2, (7) elution 3.

When isolating the plasmid DNA, several buffers were added to prepare the bacterial lysate before adding the nanoparticles for binding. In order to inhibit the function of nucleases, ET buffer containing EDTA was added to the bacterial pellet. Later the pellet was treated with RNAse and freshly prepared lysis buffer containing NaOH and SDS. A solution of KAc was added to renature only the circular plasmid DNA. At this point, all single stranded cellular DNA can be separated by precipitation and the supernatant with circular plasmid DNA can be collected [1,3]. To the collected supernatant, PEI-IONPs were added and elution was carried out using elution buffer 3 (optimized buffer system for plasmid DNA elution) at 60°C. The obtained gel picture for the eluted plasmid DNA is given in figure 7.

As depicted in figure 7, there was no DNA band for the supernatant after binding DNA, implying that all the DNA in bacterial cell culture has been bound to the nanoparticles [30]. There were no bands for the washings indicating that the bound DNA has not been eluted during the washings [30]. A clear DNA band obtained for the elution 1 showed that the PEI-IONPs have the potential to isolate plasmid DNA directly from a bacterial cell culture [31,32].

#### 3.4.2. Isolation of genomic DNA from a human blood sample

After removing the proteins and other cell debris of fresh human blood sample, the supernatant which contained genomic DNA was used to bind with PEI-IONPs. The elution of bound DNA was then carried out using elution buffer 4 (optimized buffer system for genomic DNA elution) at 60°C and the obtained results are given in electronic supplementary material, figure S9.

According to electronic supplementary material, figure S9, no DNA band can be seen for the supernatant after binding DNA, similar to the plasmid DNA bound situation, indicating that the entire DNA in the blood sample has been bound with nanoparticles. There is a very clear DNA band for elution 1 implying that most of the bound DNA has been eluted in elution 1. This clearly reflects the potential of isolating genomic DNA from a human blood sample using these PEI-IONPs as an alternative to the phenol chloroform extraction [20,33].

### 3.5. Downstream applications of isolated DNA

#### 3.5.1. Polymerase chain reaction (PCR) for isolated genomic DNA

**Figure 8.**
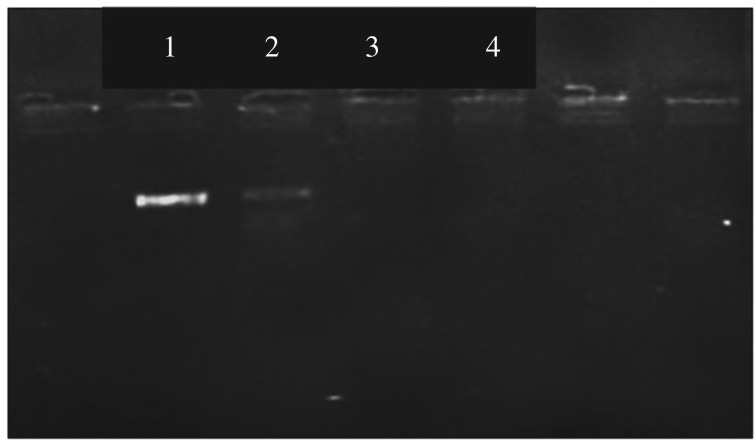
Gel picture of the PCR for isolated genomic DNA (1) marker (500 bp), (2) test sample 2, (3) test sample 1, (4) negative control.

A polymerase chain reaction (PCR) which is a very common downstream application in molecular biology was carried out for the isolated genomic DNA as explained in §2.4.1. The resulting gel picture is shown in figure 8.

The region of exon 3 in IRF 6 gene was amplified in this particular experiment. According to the gel picture, a DNA band was obtained only for the test sample 2 which contained the supernatant after elution. The fragment used for amplification was 560 bp in size and therefore 500 bp marker was loaded to identify the amplified product. In the gel picture, it was clearly seen that the DNA band corresponding to the amplified product was located slightly above compared with the DNA band corresponding to the marker. Therefore, it was shown that the desired fragment has been amplified successfully.

DNA bands were observed neither for negative control nor the test sample 1. The negative control was done without the template to check whether there were any contaminations. Since a DNA band was not obtained for that, it was clear that there was no contaminated DNA in the mixture. Test sample 1 contains the supernatant before adding PEI-IONPs. It contains genomic DNA as well as other debris present in human blood. Therefore, the absence of a DNA band for test sample 1 may be due to the lower purity. However, after the elution with PEI-IONPs, a clear DNA band can be seen as shown in test sample 2. According to these results, it was clearly proved that the magnetic separation method used in this study offers a great potential to isolate DNA with high purity and better quality.

#### 3.5.2. Magnetofection of enhanced green fluorescence protein (EGFP) into HEp-2 cells

**Figure 9.**
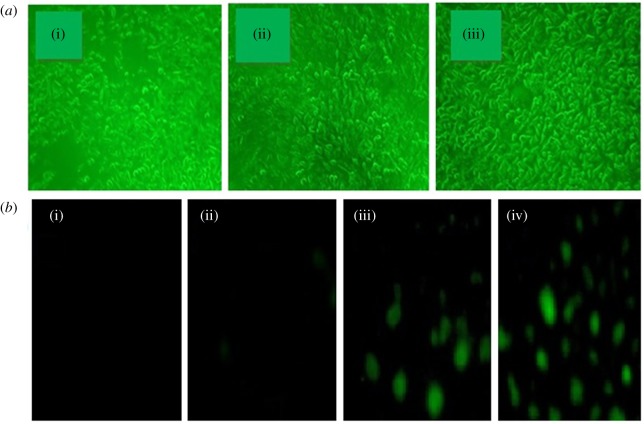
(*a*) Optical microscopic images of (i) cells transfected with PEI and DNA, (ii) cells transfected with PEI/iron oxide nanoparticles and DNA, (iii) untransfected cells, (*b*) fluorescence microscopic images of (i) untransfected cells, (ii) cells transfected with PEI and DNA, (iii), (iv) cells transfected with PEI/iron oxide nanoparticles and DNA.

The transfection procedure was carried out for the plasmid DNA isolated using PEI-IONPs as mentioned in §2.4.2. The expression of EGFP gene was studied via light and fluorescence microscopy. The results obtained are given in figure 9 and electronic supplementary material, figure S10. When only PEI and DNA were used, as in the conventional method [3], the cell growth was inhibited and a fairly high amount of cells died as shown in figure 9*a*(i). But when PEI coated iron oxide nanoparticles were used to deliver the gene, there was a considerable growth showing even formation of huge colonies as shown in figure 9*a*(ii). Figure 9*a*(iii) corresponds to the untransfected cells showing an overgrowth of cells. The results show that the cytotoxic effects of PEI [34–36] are minimized when it is delivered by binding to the surface of iron oxide nanoparticles.

In addition to light microscopy, fluorescence images were also captured after 24 h of incubation. The cells were incubated for 24 h and then the expression of EGFP protein was monitored using the fluorescence microscope. The figure 9*b* represents the untransfected sample and where no expression of EGFP protein was observed. The gene introduced with PEI alone showed a very faint fluorescence due to the low expression, as shown in figure 9*b*(ii). This is because, when PEI is used alone with DNA, at least 4 h are required for the complete transfection to take place [37,38]. But the cells were allowed to take PEI/DNA complexes only for 15–20 min, since the medium was changed after 20 min. Therefore, it could be a reason for not having an effective transfection when PEI was used alone to deliver DNA. But when PEI-IONPs were used, as given in figure 9*b*(iii)(iv), very high percentage of expression of EGFP protein can be observed. This highlighted that the DNA bound to PEI-IONPs has been effectively delivered to the cells within a short period of 15–20 min, due to the magnetically guided force provided on DNA which was obtained by PEI-IONPs. Polyethyleneimine was considered as the gold benchmark among the other transfection agents [7]. It has the ability to bind and to condense DNA into polyplexes due to the large number of amine groups present in its structure [7]. Also, it has been found that PEI is having high transfection efficiency similar to viral vectors [39]. But the problem with polyethyleneimine is, for a successful transfection to take place it requires higher amount of PEI to be delivered along with DNA which could give rise to cytotoxic effects [40]. Due to this reason it has limited the application of PEI *in vivo* studies [41]. Endosomal escaping ability of PEI is another important feature. When these types of polyplexes are taken into the cells via endocytosis, an endosome is being formed. If the cationic polymer does not have the ability to break the endosomal membrane and release the DNA to the target site, before the endosome is being matured and getting attacked by the lysozymes then the DNA is more likely to get degraded. But PEI is having very high buffering capacity due to the higher amount of amine groups present in its structure. It gives rise to huge proton accumulation which is in turn followed by passive intake of Cl^−^ ions, which will ultimately end up with osmotic swelling and endosomal membrane breakage. So this protects DNA from degradation [42].

## 4. Conclusion

In this study, PEI-IONPs were used to isolate DNA directly from biological samples. The optimized condition for plasmid DNA elution was a buffer with 0.1 M Tris HCl (pH 10.0), 1.5 M NaCl and 5% formamide, maintained at the temperature of 60**°**C. The optimized condition for genomic DNA elution was a buffer with 0.1 M Tris HCl (pH 10.0), 1.5 M NaCl and 10% formamide, maintained at the temperature of 60**°**C. According to the reported results, DNA isolated from biological samples can be directly used in downstream applications such as PCR and magnetofection. Therefore, this DNA isolation method exhibits a lot of advantages, such as significant time savings, higher yields and better purification efficiencies, compared with the other conventional DNA isolation methods. Moreover, the expression of EGFP gene in HEp-2 cells via magnetofection confirmed that these directly coated nanoparticles have enormous ability to deliver genes into eukaryotic cells under an external magnetic field. Also, it is very clear that this method of using PEI functionalized magnetic nanoparticles has drastically increased the gene transfection efficiency by minimizing the cytotoxic effects of PEI and reducing the incubation time from 4 h to 15–20 min.

## Supplementary Material

Optimized conditions for PCR and Gel Pictures

## Data accessibility

This article does not contain any additional data.
