# Supplementary material for: Magnetofection and isolation of DNA using polyethyleneimine functionalized magnetic iron oxide nanoparticles
Source: R Soc Open Sci. 2018 Dec 12;5(12):181369. doi: 10.1098/rsos.181369 (PMC6304157; doi:10.1098/rsos.181369)
Supplement: Optimized conditions for PCR and Gel Pictures [file rsos181369supp1.docx]

**Magnetofection and isolation of DNA using Polyethyleneimine Functionalized Magnetic Iron Oxide Nanoparticles**

Adheesha N. Danthanarayana^1^, Danushika C. Manatunga^1^, Rohini M. de Silva^1*^, N. Vishvanath Chandrasekharan^1^ and K. M. Nalin de Silva^1,2^

**Supplementary Information**

Table S1: Optimized conditions for PCR

| Action | Temperature | Time (seconds) |
| --- | --- | --- |
|  | ( ºC) |  |
| Initial denaturation | 94 | 120 |
| Denaturation | 94 | 30 |
| Annealing | 57 | 30 |
| Extension | 72 | 30 |
| Post extension | 72 | 420 |


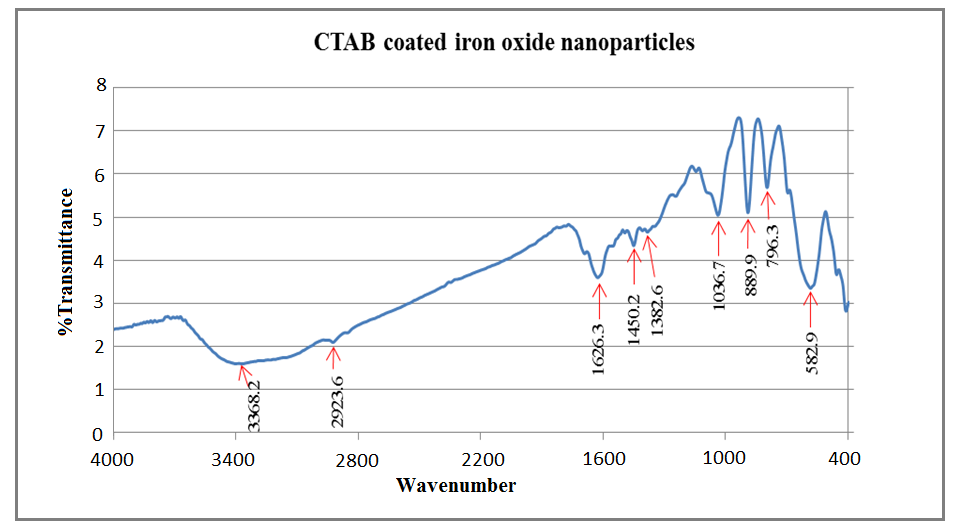


**Figure S1.** FT-IR spectrum of iron oxide nanoparticles coated with CTAB


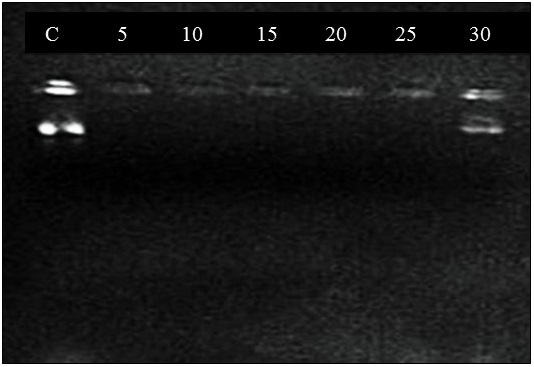


**Figure S2**. Gel picture of the remaining DNA in the supernatant in the process of finding the DNA binding capacity (C-control, 5-30 µg)


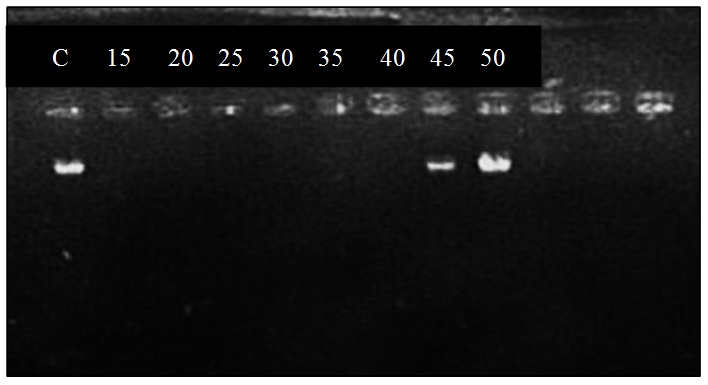


**Figure S3.** Gel picture of the remaining DNA in the supernatant in the process of finding the DNA binding capacity (C-control)


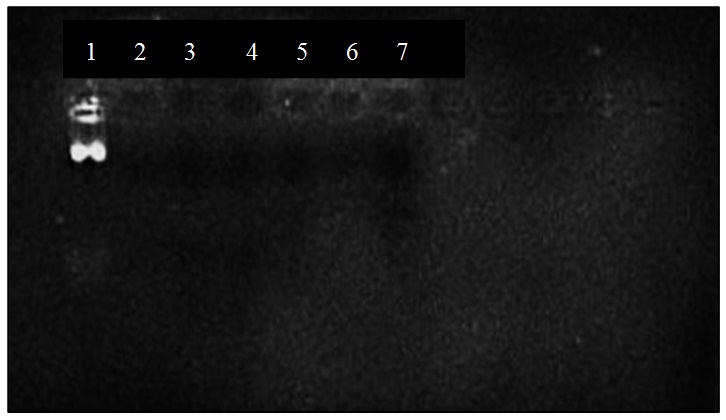


**Figure S4.** Gel picture of plasmid DNA elution with elution buffer 1, (1) Control (2) Supernatant after binding DNA (3) Washing 1 (4) Washing 2 (5) Elution 1 (6) Elution 2 (7) Elution 3


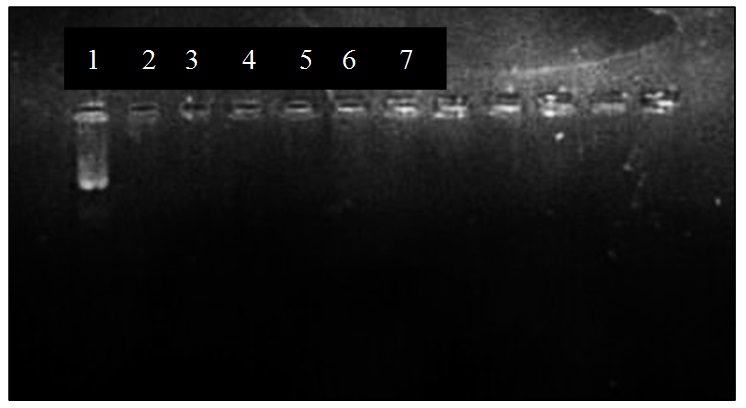


**Figure S5.** Gel picture of plasmid DNA elution with elution buffer 1 heated up to 60 º (1) Control (2) Supernatant after binding DNA (3) Washing 1 (4) Washing 2 (5) Elution 1 (6) Elution 2 (7) Elution 3


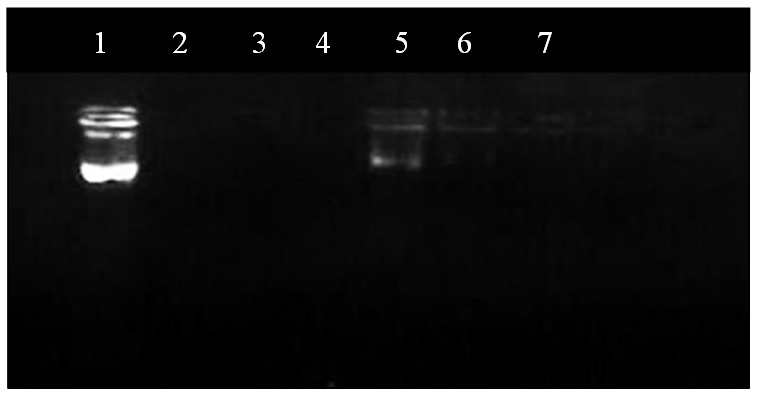


**Figure S6**. Gel picture of plasmid DNA elution with elution buffer 2 heated up to 60 ºC (1) Control (2) Supernatant after binding DNA (3) Washing 1 (4) Washing 2 (5) Elution 1 (6) Elution 2 (7) Elution 3


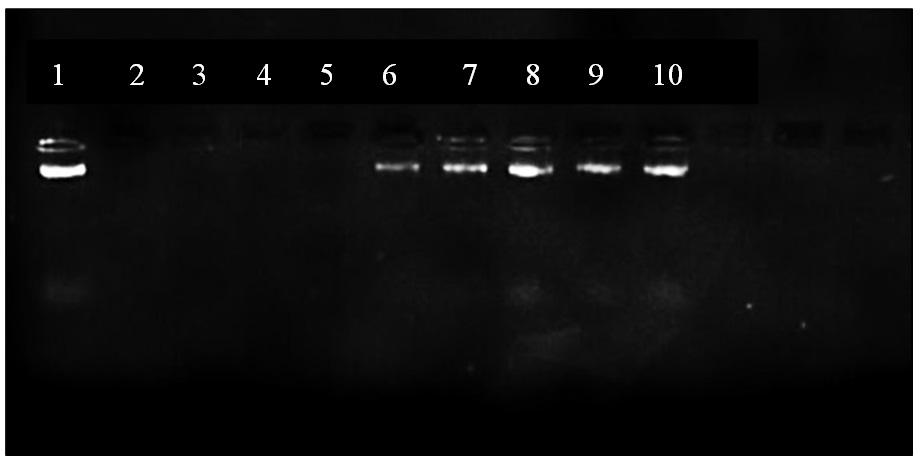


**Figure S7.** Gel picture of plasmid DNA elution with elution buffer 3 at different temperatures (1) Control (2) Supernatant after binding DNA (3) Washing 1(4) Washing 2 (5) Elution at 30 ºC (6) Elution at 50 ºC (7) Elution at 55 ºC (8) Elution at 60 ºC (9) Elution at 65 ºC (10) Elution at 70 ºC


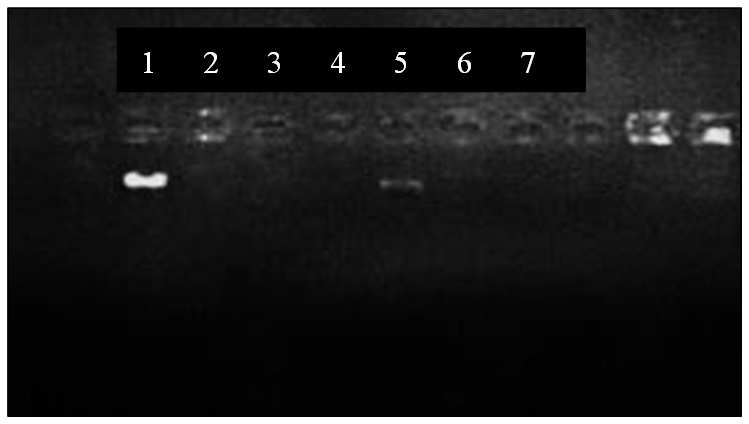


**Figure S8**. Gel picture of genomic DNA elution with elution buffer 3 heated up to 60 ºC (1) Control (2) Supernatant after binding DNA (3) Washing 1 (4) Washing 2 (5) Elution 1 (6) Elution 2 (7) Elution 3


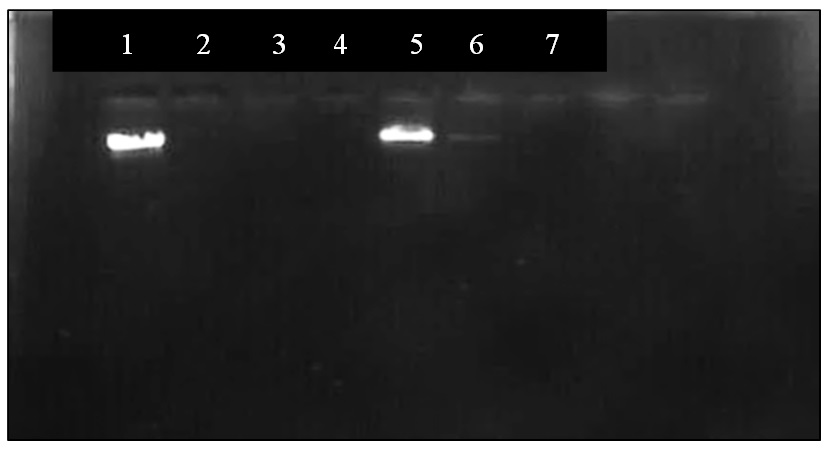


**Figure S9.** Gel picture of the genomic DNA eluted using PEI-IONPs from a human blood sample (1) Control (2) Supernatant after binding DNA (3) Washing 1 (4) Washing 2 (5) Elution 1 (6) Elution 2 (7) Elution 3
